# Pancreas Cancer Survival in the Gemcitabine Era

**DOI:** 10.4137/cmo.s334

**Published:** 2008-04-29

**Authors:** Mitchell S. Wachtel, K. Tom Xu, Yan Zhang, Maurizio Chiriva-Internati, Eldo E. Frezza

**Affiliations:** 1 Department of Pathology, Texas Tech University Health Sciences Center, Lubbock Texas; 2 Department of Family and Community Medicine, Texas Tech University Health Sciences Center, Lubbock Texas; 3 Department of Microbiology and Immunology, Texas Tech University Health Sciences Center, Lubbock Texas; 4 Department of Surgery, Texas Tech University Health Sciences Center, Lubbock Texas

**Keywords:** gemcitabine, pancreas cancer, survival, metastatic disease

## Abstract

After multiple positive studies, gemcitabine, approved for the treatment of pancreas cancer by the FDA in 1977, became standard of care. Whether this therapeutic advance has translated into longer survival for pancreas cancer patients in general has not been established. This study, derived from SEER (Surveillance, Epidemiology, and End Results (SEER) Program of the National Cancer Institute) data, compared the survival experiences of the gemcitabine (1998–2004) and pre-gemcitabine (1988–1997) eras for 7,151 patients who had metastatic disease and did not undergo extirpative surgery, 14,369 patients who had not undergone surgery and had metastases, 5,042 patients who had undergone surgery and did not have metastases, and 5,011 patients who had undergone surgery and had metastases. Calculated survival time ratios (TR) were adjusted for radiotherapy history, grade, nodal status, loco-regional extent of disease, age, race, and gender. For those who did not undergo extirpative surgery, improvements in survival in the gemcitabine era (1998–2004) versus the prior time period (1988–1997) seen for patients with metastatic cancer (TR = 1.20, 95% c.i. 1.15–1.25) were not seen for those without metastatic cancer (TR = 1.05, 95% c.i. 1.00–1.15). For those who did undergo extirpative surgery, improvements were much more dramatic for those with metastatic cancer (TR = 1.61, 95% c.i. 1.45–1.80) than those without metastases (TR = 1.23, 95% c.i. 1.15–1.31). The results are consistent with the notion that the promising findings with respect to gemcitabine in the controlled clinical trials have found expression in the general population of patients with pancreas cancer.

## Background

Pancreas cancer is a common cancer that is commonly lethal, being the fourth leading site for cancer deaths for both men and women. (Jemal, Siegel et al. 2008) After the results of Burris, et al., (Burris, Moore et al. 1997) were published, FDA approval in 1997 for gemcitabine as a chemotherapeutic agent to treat metastatic pancreas cancer followed. Studies supporting the efficacy of this agent (Rothenberg, Moore et al. 1996; Burris and Storniolo, 1997; Burris, Moore et al. 1997; Rothenberg, Sharma et al. 1998; Tempero, Plunkett et al. 2003; Burris, 2005; Ko, Dito et al. 2006; Gansauge, Ramadani et al. 2007) proved so positive that single agent gemcitabine therapy is now standard of care, (Burris, 2005) rendering justified the designation of the years 1988–2007 as the gemcitabine era. Whether this therapeutic advance has translated into longer survival for pancreas cancer in general, however, has not been established. We hypothesized that analyzing survival differences between the gemcitabine and the pre-gemcitabine era might shed light on this question, provided separate analyses were performed on patients with and without metastases, separating patients into those who had and had not undergone surgery. As a source of general information concerning cancer patients in the United States, the Surveillance, Epidemiology, and End Results (SEER) Program of the National Cancer Institute is unparalleled because it collects and publishes cancer incidence and survival data from 14 population-based cancer registries and three supplemental registries covering approximately 26% of the US population. (National Cancer Institute, 2008).

This study compared survival during the gemcitabine era with that of the pre-gemcitabine era for evaluated for 7,151 patients metastatic disease and did not undergo extirpative surgery (group 1), 14,369 patients who had not undergone surgery and had metastases (group 2), 5,042 patients who had undergone surgery and did not have metastases (group 3), and 5,011 patients who had undergone surgery and had metastases (group 4). Survival ratios were adjusted for an array of potentially confounding variables. Results of the analysis were then used to subdivide the study population to provide empirical median survival estimates for the gemcitabine era stratified by surgical status, the history of radiotherapy, and nodal status.

## Materials and Methods

A case listing session was performed using the Surveillance and End Results 17 Registries Limited-Use, November 2006 sub (1973–2004 varying) database. Patients were excluded if they were recorded as having been dead or lost to follow-up at the time of diagnosis, if the tumor was not microscopically confirmed, if it was not known whether or not the patient had metastatic tumor, if the behavior was not listed as being malignant, if it was not known whether or not the patient had had surgery, if the patients were of unknown race or age, if the ICD code was other than 8140, 8141, 8480, 8481, or 8489, if the site of origin was listed as being other than as listed below, if the diagnosis was rendered before 1988, or if a prior cancer had been diagnosed. Explanatory variables considered were: 1) surgery—extirpative surgery versus not; metastatic disease—present versus absent; year of diagnosis: after gemcitabine approval (1998–2004) versus before (1988–1997); 2) radiation therapy—provided versus not provided versus unknown; 3) grade—high (grades III and IV) versus low (grades I and II) versus ungraded; 4) loco-regional extent—confined to pancreas versus extended beyond pancreas versus unknown; 5) nodal status—positive versus negative versus unknown; 6) origin within pancreas—head versus body, tail, or overlapping versus pancreas, not otherwise specified; 7) age—at or above median 68 years old versus younger; 8) race—White versus Black versus Other; and 9) gender.

The intent was to determine if the effect of gemcitabine might be evident in survival patterns of patients with pancreas cancer. To this end, four separate analyses calculated adjusted survival time ratios for each of the subsets, group 1, group 2, group 3, and group 4. Cox proportional hazards and Rayleigh, lognormal, loglogistic, Weibull, and exponential accelerated failure time models were considered as means to analyze survival experience. Because lognormal regression had the lowest overall AIC (Aikake’s an Information Criterion) value, it was used to estimate adjusted ratios of survival times and 95% confidence intervals. Log rank tests were used for univariate comparisons. Kaplan-Meier methods were used to calculate median survivals and 95% confidence intervals. Null hypotheses were rejected when *P* < 0.05. All analyses were performed on R.

## Results

Of 31,573 patients retrieved, 28,894 (91.5%) died, with a median survival of five months. Table 1 displays distributions of patients and deaths, as well as median survivals, stratified by the predictor variables. Log rank tests showed that each grouping demonstrated differences with respect to survival that could not have been explained by chance.

**Figure 1:**
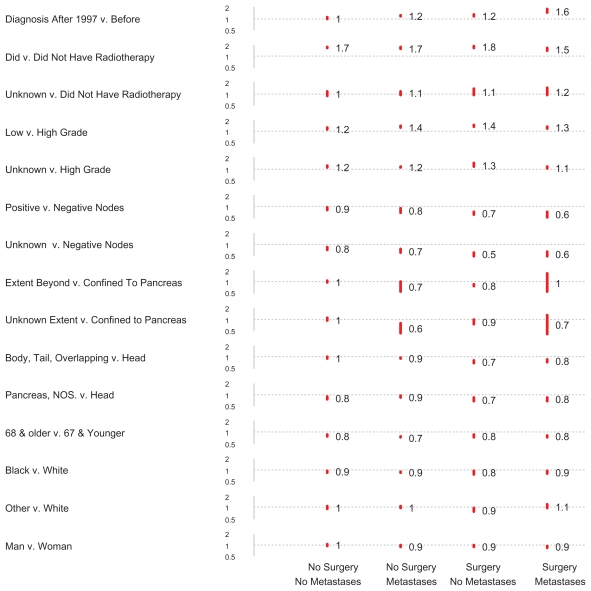
Adjusted survival time ratios and 95% confidence intervals (red bars), as calculated by log normal regression, for 31,573 patients with pancreatic adenocarcinoma. Each column represents the results of a regression analysis in which the variables evaluated for their effects on survival included year of diagnosis, radiotherapy, grade, nodal status, loco-regional extent, origin within the pancreas, age, race, and gender.

Figure 1 shows adjusted survival time ratios (TR) and 95% confidence intervals (red bars) as calculated by log normal regression. Each column displays all comparisons of a separate analysis: the leftmost shows results for 7,151 patients in group 1; the second, for 14,369 in group 2; the third, for 5,042 in group 3; and the fourth, for 5,011 in group 4. Each row shows results for all four groups for a comparison. Note the dotted grey lines; where these cross red bars, as is true for loco-regional extent comparisons for patients in group 4, results are not statistically significant (*P* > 0.05).

For those who did not undergo extirpative surgery, improvements in survival in the gemcitabine era (1998–2004) versus the prior time period (1988–1997) seen for patients with metastatic cancer (group 2) (TR = 1.20, 95% c.i. 1.15–1.25) were not seen for those without metastatic cancer (group 1) (TR = 1.05, 95% c.i. 1.00–1.15). For those who did undergo extirpative surgery, improvements were much more dramatic for those with metastatic cancer (group 4) (TR = 1.61, 95% c.i. 1.45–1.80) than those without metastases (group 3) (TR = 1.23, 95% c.i. 1.15–1.31).

Compared with those who had not received radiation therapy, those who did had much longer median survivals; the lower limits of all four 95% confidence intervals were above 1.5; radiotherapy was associated with a survival time increase of over 50%. As expected, having one or more lymph nodes with metastatic cancer yielded a shorter survival than having no lymph nodes with meta-static cancer. Patients without lymph node sampling had an even shorter survival than those who had negative lymph nodes.

With respect to extent of disease, differences with respect to the presence or absence of metastases were quite prominent. Whereas only 12% of patients without metastases lacked information about loco-regional spread, 89% of patients with metastases did. Only 37 (0.2%) of patients with metastatic disease were recorded as having disease confined to the pancreas. With such numbers, it is unsurprising that confidence intervals for patients with metastases were so wide as to preclude definite assertions about the importance of confinement or lack of confinement of the tumor to the pancreas. By contrast, for patients without metastases adjusted survival differences with respect to local extent of disease were definitely small. Apart from the above discussed variables, remaining TR point estimates were modest, ranging from 0.73 to 1.43.

**Figure 2:**
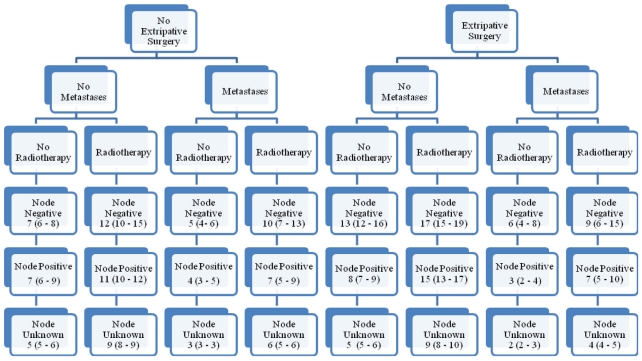
Empirical median survivals (95% confidence intervals) for 12,543 patients with pancreatic adenocarcinoma diagnosed from 1988–1997.

**Figure 3:**
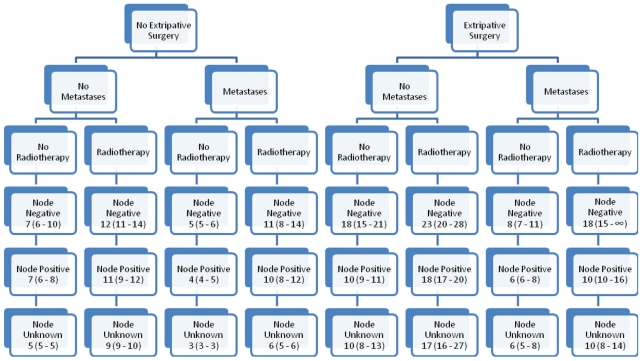
Empirical median survivals (95% confidence intervals) for 19,030 patients with pancreatic adenocarcinoma diagnosed from 1998–2004.

Attention was directed to differences with respect to surgical history, metastases, radiation therapy, lymph node status, and the gemcitabine era. Figure 2 displays empirically derived median survivals in months for the years 1988–1997; Figure 3, for the years 1998–2004. Results were consistent with prior analyses. Consider the differences between eras: median survival point estimates for group 1 hardly changed; that for groups 2 and 3 showed modest increments; that for group 4 showed dramatic increments. Among patients with negative or unknown nodal status, median survival point estimates for those who underwent surgery were greater than for those who did not. For positive nodes, results with respect to surgery were varied. Uniformly, radiotherapy was associated with larger and metastatic disease with smaller median survival point estimates. For the most part, patients with negative nodes had larger median survival point estimates than did those with positive nodes, who in turn had larger median survival point estimates than those with unknown nodal status.

## Discussion

The results are consistent with the notion that the promising findings with respect to gemcitabine in the controlled clinical trials have found expression in the general population of patients with pancreas cancer. Adjusted for radiotherapy history, grade, nodal status, loco-regional extent of disease, age, race, and gender, no improvement was seen in patients without metastatic disease who did not undergo surgery. With the same adjustments, the improvement among surgically treated patients was greater for those who had metastatic disease than for those who did not. Because radiotherapy tends to be given palliatively, the general improvement in survival accorded patients who underwent radio-therapy suggests a survival benefit that extends beyond a negative selection bias. Finally, a wide array of complex interactions exists among factors that are shown to affect survival, especially with respect to the benefit of surgery.

After the seminal work of Burris, et al., (Burris, Moore et al. 1997) Food and Drug Administration approval was granted to gemcitabine for the treatment of metastatic pancreas cancer. A wide array of clinical trials (Rothenberg, Moore et al. 1996; Burris and Storniolo, 1997; Burris, Moore et al. 1997; Rothenberg, Sharma et al. 1998; Tempero, Plunkett et al. 2003; Burris, 2005; Ko, Dito et al. 2006; Gansauge, Ramadani et al. 2007) have proven the efficacy of this chemotherapeutic agent, such that gemcitabine’s use as single agent chemotherapy for metastatic pancreas cancer is now standard of care. (Burris, 2005) For this reason, it is reasonable to ask whether or not the use of this agent has been associated with a general increment in survival among patients with metastatic pancreas cancer. The results of this study provide an affirmative answer to that question. Patients who would be unlikely to benefit from gencitibine, those without extirpative surgery or metastatic disease, failed to show an increment in survival time, when results were adjusted for other variables. By contrast, patients with metastatic surgery who had not undergone surgery, who would be candidates for this agent, showed about a 20% increase in survival, similar to that experienced by patients without metastatic cancer who had undergone surgery; advances in chemotherapy have, in this case, matched advances in surgical technique in terms of advancing patient survival. The Will Rogers effect may be partly responsible for the increased life expectancy seen in patients who underwent surgery and did not have metastatic disease. (Basu and Alavi, 2008) This criticism does not apply pari passu to patients with metastatic cancer; the 60% increment in survival seen in patients who had undergone surgery and had metastatic cancer, about 40% more than the increment associated with surgery alone, occurred within the context of a group’s being thought to be in an equally bad state of affairs before or after the Will Roger’s effect with respect to pre-operative studies had taken place. Moreover, the lack of a survival increment for patients who had neither metastatic disease nor surgery militates against the existence of a Will Roger’s effect.

The relationship of surgery to survival is complex. An array of studies have documented the efficacy, safety, and underutilization of surgical treatment of pancreas cancer. (Imamura, Doi et al. 2004; Lygidakis, Singh et al. 2004; Fesinmeyer, Austin et al. 2005; Fong, Gonen et al. 2005; Lygidakis, Jain et al. 2005; Mu, Peng et al. 2005; Kunisaki, Akiyama et al. 2006; Bilimoria, Bentrem et al. 2007; Bilimoria, Bentrem et al. 2007; Finlayson, Fan et al. 2007; Hirano, Kondo et al. 2007; Lygidakis, Sharma et al. 2007; Reddy, Tyler et al. 2007; Riall and Lillemoe, 2007; Ryska, Strnad et al. 2007; Bruzoni, Johnston et al. 2008; Sa Cunha, Rault et al. 2008; Westgaard, Tafjord et al. 2008) One randomized, controlled trial showed that, for localized pancreas cancer involves neither superior mesenteric nor common hepatic arteries, (Imamura, Doi et al. 2004) surgery was superior to chemoradiotherapy. A second trial showed that, for patients with advanced pancreatic head cancer with vascular invasion who underwent chemoimmuno-therapy, (Lygidakis, Singh et al. 2004) resection was superior to palliative bypass. Far fewer resection procedures are being performed than is currently warranted. (Bilimoria, Bentrem et al. 2007; Riall and Lillemoe, 2007) Although safety issues arise with respect to older patients (Finlayson, Fan et al. 2007) and to hospitals that infrequently perform resections, (Fong, Gonen et al. 2005) the safety, in general, of extirpative surgery is well-established. (Riall and Lillemoe, 2007) One study found surgery the most important survival factor. (Fesinmeyer, Austin et al. 2005) The difficulty with crediting surgical extirpation its survival advantage lies in the selection bias; patients are excluded from surgical extirpation if metastatic disease is known to be present, if the tumor does extensively invades blood vessels, and if the Karnovsky score is below 70, (Wolff, Abbruzzese et al. 2008) meaning those who do undergo surgery are likely to do better. This study’s findings raises the possibility that surgery’s advantage depends upon lymph node status moreso than it did on metastatic spread or radiotherapy; since the principal interest of this study lay in the effect of gemcitabine, this suggestion should be evaluated in a separate analysis. If this suggestion is confirmed, the contraindication to surgical extirpation by limited metastatic disease might be re-considered.

With respect to radiotherapy, results of clinical trials have been mixed, (1987; Klinkenbijl, Jeekel et al. 1999; Neoptolemos, Stocken et al. 2004; Smeenk, de Castro et al. 2005; Stocken, Buchler et al. 2005; Garofalo, Regine et al. 2006; Sultana, Neoptolemos et al. 2006; Saif 2007; Smeenk, van Eijck et al. 2007; Artinyan, Hellan et al. 2008) with divergent opinions concerning the same study being published. (Klinkenbijl, Jeekel et al. 1999; Garofalo, Regine et al. 2006) For this reason, radiotherapy continues to be viewed as an option to be reserved for research protocols and specialized centers. (Wolff, Abbruzzese et al. 2008) Notwithstanding these reservations, the present study found differences in survival with respect to all subgroups, confirming and extending the findings of a very recent study of node negative pancreas cancer using the same database. (Artinyan, Hellan et al. 2008) Part of the difficulty in interpreting these results stems from a limitation of the Surveillance and End Results Database, the absence of information regarding the concomitant administration of chemotherapy, also noted in the prior study. (Artinyan, Hellan et al. 2008) For this reason, until the benefit of radiotherapy is settled by clinical trials, radiotherapy should perhaps best be viewed in studies of patient databases as a variable to be taken into consideration in analyses of survival undertaken for other reasons.

Apart from its having lower absolute adjusted TR values than radiotherapy or nodal status, grade was not chosen for purposes of stratifying the sample into subgroups because there exists no consensus at this time as to which of the multiple grading systems for pancreas cancer should be used. (Cubilla, Fitzgerald et al. 1978; Greene, Page et al. 2002; Brennan, Kattan et al. 2004; Adsay, Basturk et al. 2005; Compton, 2005; Eltoum, Eloubeidi et al. 2005; Ferrone, Kattan et al. 2005; Johnson and Adamo, 2007) Although the original four grade system is still recognized by the American Joint Committee on Cancer, (Greene, Page et al. 2002) Surveillance Epidemiology and End Results, (Johnson and Adamo, 2007) and the College of the American Pathologists, (Compton, 2005) many do not use this system, preferring instead to use a three tiered system. (Brennan, Kattan et al. 2004; Ferrone, Kattan et al. 2005) The three tiered system, however, does have its flaws, one of which is that at least one study shows that moderately differentiated tumors have a better prognosis than do well differentiated tumors; (Adsay, Basturk et al. 2005) the latter finding was one of the principle reasons grade 1 and grade 2 tumors were combined into a low grade category. Perhaps the multiplicity and complexity of grading systems in part induced the lack of a grade for half the pancreas cancer patients. A two category grading system has been shown to be an effective adjunct to the cytologic diagnosis of pancreas cancer; (Eltoum, Eloubeidi et al. 2005) a two grade system should also be considered for histologic material. (Adsay, Basturk et al. 2005)

Limitations of database analyses have been previously described, (Artinyan, Hellan et al. 2008) but bear some repetition. As opposed to database analyses, in clinical trials (Pocock, 1983) one set of pathologists reviews slides, one set of radiologists reviews radiographs, one set of surgeons performs surgery, one set of chemotherapists provides chemotherapy, and one set of radiation oncologists provides radiation therapy. Protocols are carefully followed, with deviations oft resulting in exclusions from the study. Patients are usually carefully selected to define groups being compared. Unfortunately, as is true of assessments of radiation therapy in pancreas cancer, (Klinkenbijl, Jeekel et al. 1999; Neoptolemos, Stocken et al. 2004; Garofalo, Regine et al. 2006) results of controlled clinical trials contradict one another. Meta analyses (Sutton, Abrams et al. 2000; Stocken, Buchler et al. 2005) can be performed as a means to summarize expert opinion and the results of published data; by definition such analyses suffer to some degree from the limitations of population databases. Clinical trials and meta analyses can not completely reflect the world at large, where patients are treated by a wide array of physicians and rarely fall into carefully defined groups. Additional studies should evaluate the experience of patients in other nations. Because median survival of pancreas cancer patients is less than one half of a year, better analyses would result from studies that record the number of weeks and not months of survival. Most important, data concerning chemotherapy administered to patients would greatly assist the analysis of their survival. This is a clinical epidemiology study, not a clinical study in which clinical parameters can be better controlled among a much smaller number of patients. The gain from using this data set with limited clinical information lies in its representativeness and standardization. The above limitations notwithstanding, database analyses permit one to see if benefits of carefully controlled studies have found their way into the population of patients at large; the present study provides some confirmation that gemcitabine effected increases in survival of pancreas cancer patients with metastases.

In summary, pancreas cancer patients with neither metastases nor a history of surgery fared no better in the gemcitabine era than before, in contrast to those with metastases who had not undergone surgery. Among patients who had undergone surgery, those who had metastases showed greater improvements in survival than those who did not. Taken together, the results provide evidence for the efficacy of gemcitabine as single agent therapy for metastatic pancreas cancer.

## Figures and Tables

**Table 1:** Characteristics of interest of pancreatic adenocarcinoma patients. For all log rank tests, *P* < 0.01.

	Patients	Deaths	Survival in months	Log rank test

	(n = 31,573)	(n = 28,894)	Median (95% c.i.)	χ^2^ (d.f.)
Extirpative surgery				
No	21,520 (68.2%)	19,918 (68.9%)	4 (4–4)	
Yes	10,053 (31.8%)	8,976 (31.1%)	6 (6–6)	1,040 (1)
Metastases				
No	12,193 (38.6%)	10,523 (36.4%)	9 (9–9)	
Yes	19,380 (61.4%)	18,371 (63.6%)	3 (3–3)	4,794 (1)
Year of diagnosis				
1988–1997	12,543 (39.7%)	12,372 (42.8%)	4 (4–4)	
1998–2004	19,030 (60.3%)	16,522 (57.2%)	5 (5–5)	98 (1)
Radiotherapy				
No radiotherapy administered	23,951 (75.9%)	22,289 (77.1%)	4 (4–4)	
Radiotherapy administered	7,059 (22.4%)	6,078 (21.0%)	10 (9–10)	
Unknown	563 (1.8%)	527 (1.8%)	5 (5–6)	2,460 (2)
Grade				
High (III or IV)	7,231 (22.9%)	6,731 (23.3%)	4 (4–4)	
Low (I or II)	8,473 (26.8%)	7,508 (26.0%)	7 (7–7)	
Unknown	15,869 (50.3%)	14,655 (50.7%)	4 (4–4)	1,142 (2)
Nodal status				
Negative	2,775 (8.8%)	2,215 (7.7%)	12 (12–13)	
Positive	4,283 (13.6%)	3,676 (12.7%)	9 (9–10)	
Unknown	24,515 (77.6%)	23,003 (79.6%)	4 (4–4)	3,469 (2)
Loco-regional extent				
Confined to pancreas	2,752 (8.7%)	2,471 (8.6%)	9 (9–9)	
Beyond pancreas	10,097 (32.0%)	9,458 (32.7%)	8 (8–8)	
Unknown	18,724 (59.3%)	16,965 (58.7%)	3 (3–3)	4,013 (2)
Origin				
Head	17,159 (54.3%)	15,386 (53.2%)	6 (6–6)	
Body, Tail, Other	8,798 (27.9%)	8,169 (28.3%)	4 (4–4)	
Pancreas, NOS	5,616 (17.8%)	5,339 (18.5%)	3 (3–3)	1,286 (2)
Age				
68 or Older	15,302 (48.5%)	13,742 (47.6%)	6 (6–6)	
67 or Younger	16,271 (51.5%)	15,152 (52.4%)	4 (4–4)	470 (1)
Race				
White	25,529 (80.9%)	23,349 (80.8%)	5 (5–5)	
Black	3,669 (11.6%)	3,410 (11.8%)	4 (4–4)	
Other	2,375 (7.5%)	2,135 (7.4%)	5 (5–5)	49 (2)
Gender				
Female	15,530 (49.2%)	14,169 (49.0%)	5 (5–5)	
Male	16,043 (50.8%)	14,725 (51.0%)	5 (5–5)	7.8 (1)
